# Photocatalytic performance of Cu_2_O-loaded TiO_2_/rGO nanoheterojunctions obtained by UV reduction

**DOI:** 10.1007/s10853-017-0911-2

**Published:** 2017-02-21

**Authors:** Kaituo Dong, Jiandong He, Junxue Liu, Fengting Li, Lianqing Yu, Yaping Zhang, Xiaoyan Zhou, Hongzhang Ma

**Affiliations:** 1 College of Science, China University of Petroleum, Qingdao, 266580 China; 2 College of Chemical Engineering, China University of Petroleum, Qingdao, 266580 China; 3 State Key Laboratory of Molecular Reaction Dynamics, Dalian Institute of Chemical Physics, Chinese Academy of Sciences, 457 Zhong Shan Rd., Dalian, 116023 China

## Abstract

A novel dot-like Cu_2_O-loaded TiO_2_/reduced graphene oxide (rGO) nanoheterojunction was synthesized via UV light reduction for the first time. Cu_2_O with size of ca. 5 nm was deposited on rGO sheet and TiO_2_ nanosheets. The products were characterized by infrared spectroscopy, Raman spectrum, UV–Vis diffuse reflectance spectra, XPS techniques, photoluminescence spectra. The results demonstrated that Cu_2_O and rGO enhanced the absorption for solar light, separation efficiency of electron–hole pairs, charge shuttle and transfer, and eventually improved photoelectrochemical and photocatalytic performance for contaminants degradation. The reaction time and anion precursor could affect the final copper-containing phase. As extending UV irradiation time, Cu^2+^ was be first reduced to Cu_2_O and then transformed to metal Cu. In comparison with CH_3_COO^−^ (copper acetate), NO_3_^−^ (copper nitrate) and Cl^−^ (copper chloride), SO_4_^2−^ (copper sulfate) was the optimum for synthesizing pure Cu_2_O phase.

**Electronic supplementary material:**

The online version of this article (doi:10.1007/s10853-017-0911-2) contains supplementary material, which is available to authorized users.

## Introduction

Exploration of an optimum semiconductor nanoheterojunction architecture for enhanced photoelectrochemical properties had been developed with great efforts for years [1–5]. Varied architectures, such as bulk crystal/bulk crystal, core/shell, bulk crystal/dotted crystal et al., had been intensively studied [3, 6, 7]. Architecture of bulk crystal/dotted crystal was similar with a component of dye-sensitized or semiconductor quantum dot-sensitized TiO_2_ in solar cell, owning high photoelectrochemical performance [8, 9]. For this architecture, dotted crystal with special structure and size had a tunable contact area on the surface of matrix [4, 10, 11]. TiO_2_ nanosheets exposing (001) facet, which had excellent photocatalytic performance, made itself a stable substrate for building TiO_2_-based heterojunctions architecture, while its wide band gap of 3–3.2 eV limited the absorption of sun light. Loading dot-like semiconductor with response of visible light on TiO_2_ nanosheets might be an optimum architecture.

Cuprous oxide (Cu_2_O) was a relative stable p-type semiconductor with direct band gap of 2.0–2.2 eV which could absorb visible light below 600 nm [12, 13]. In addition, its conduction and valence band positions matched well with those of n-type TiO_2_, which facilitated separation of photo-induced electron–hole pairs [13–15]. However, TiO_2_ nanosheet/dot-like Cu_2_O crystal heterojunction still had poor electron conductivity [16]. Reduced graphene oxide (rGO) owning graphitic *sp* ^2^ and *sp* ^3^-hybrid structures had comparable conductivity of metal and large surface area as a substrate for building heterojunctions [17, 18]. It is reported that particles of TiO_2_ or Cu_2_O combining with rGO had enhanced charge shuttle and transfer performance [13, 19, 20]. So the dot-like Cu_2_O-loaded TiO_2_/rGO nanoheterojunction might become one of the most efficient TiO_2_-based photocatalysts.

General method for loading dot-like Cu_2_O crystal on the TiO_2_ or rGO is reduction of various cupric salts with strong chemical reagents in alkaline condition at high temperature [14, 21, 22]. For example, Wang or Geng et al. [14, 23] synthesized nanocrystalline Cu_2_O on TiO_2_ frame or arrays using cupric acetate as precursor and glucose as reducing reagent. Gao et al. [24] loaded Cu_2_O particle on rGO sheet using l-ascorbic acid as reductive reagent in mild condition. Compared to chemical liquid reduction, photochemical synthesis of Cu_2_O had advantages of free chemical reagents addition, room temperature, atmospheric pressure, free of pH adjustment via alkali or acid. In previous reports, Cu_2_O was synthesized via γ-ray radiation [25, 26]. However, γ-ray radiated by ^60^Co source is very environmental unfriendly, harmful and strictly restricted by laws.

In this work, γ-ray was alternated by a ultraviolent (UV) light (main peak 254 nm, 25 W), and dot-like Cu_2_O crystal with size of ca. 5 nm was successfully deposited on TiO_2_ nanosheet/rGO. To our knowledge, this has never been reported before. The results revealed that the newly designed nanoheterojunction had strong absorption of solar light, high separation efficiency of electron–hole pairs and high performance of charge shuttle and transfer. Surfactants such as sodium dodecyl benzene sulfonate (SDBS) existing in cleaning agents, dyes such as methyl orange (MO), rhodamine B (RhB) as the aromatic-containing macromolecules existing in waste water were selected to evaluate its photocatalytic activity [27].

More importantly, various cupric salts with different anions such as SO_4_^2−^, Cl^−^, CH_3_COO^−^, NO_3_^−^ were employed to synthesize Cu_2_O in previous works [14, 28–30]. In this report, taken different stabilities, chemical activities and chelating ability with positive ion into consideration, these cupric salts as precursors were studied to explore the synthetic mechanism under photochemical condition.

## Experiment

### Materials

Natural graphite was purchased from Qingdao Baichun graphitic Co., Ltd. Fluorine tin oxide (FTO)-coated glass (resistivity <10 Ω sq^−1^) was purchased from Zhuhai Kaivo Electronic Components Co., Ltd. The other chemical reagents were purchased from Sinopharm chemical reagent Co., Ltd. And all the chemicals were used without further purification.

### Synthesis of graphite oxide

Graphite oxide was synthesized by the typical modified Hummers’ method [31]. In details, 2 g of natural graphite flakes was mixed with 1 g sodium nitrate in the ice bath. Then, 50 mL concentrated H_2_SO_4_ was slowly added into the mixture under stirring to keep temperature under 5 °C. 0.3 g potassium permanganate was slowly put into the mixture under stirring to maintain temperature below 20 °C. Then, 7 g potassium permanganate was slowly added into the mixture for 1 h to keep temperature below 20 °C. Successively, the mixed solution was stirred at 35 °C for 2 h, followed by slow addition of deionized (DI) water (90 mL). After that, the solution was heated to 98 °C and kept for 15 min. The suspension was further diluted with 55 mL DI warm water, and then, 7 mL H_2_O_2_ was added to terminate the reaction. The mixture was filtered and washed with 10% HCl (1 L) and DI water (1 L) until pH 7. The graphite oxide product was vacuum-dried at 40 °C for 12 h.

### Synthesis of TiO_2_ nanosheets

The TiO_2_ nanosheets were synthesized by hydrothermal method [32]. In a typical experimental procedure, 5 mL of tetrabutyl titanate [Ti(OBu)_4_, ≥98%] and 0.6 mL of hydrofluoric acid (HF) (≥40%) were mixed in a dried Teflon autoclave with a capacity of 20 mL, and kept at 180 °C for 24 h. The powder was separated by centrifugation, washed by water and ethanol several times, consecutively. The final product was vacuum-dried at 80 °C for 6 h. Caution! HF is extremely corrosive and a contact poison, and it should be handled with extreme care. Hydrofluoric acid solution should be stored in plastic container and used in a fume hood.

### Synthesis of TiO_2_/Cu_2_O composite

20 mg TiO_2_ nanosheets were sonicated in 100 mL ethanol for 15 min and then poured into 100 ml CuSO_4_ aqueous solution (containing 3 mmol CuSO_4_·5H_2_O) with fiercely stirring. The following procedure was the same with the Cu_2_O synthesis (shown in supporting information), and this obtained TiO_2_/Cu_2_O composite was labeled as TC.

### Synthesis of TiO_2_/rGO/Cu_2_O composites

10 mg graphite oxide was sonicated in 100 mL deionized water at 30–40 °C for 30 min to obtain clear suspension; then, 3 mmol CuSO_4_·5H_2_O was added and dissolved. The following synthesis procedure was as same as that of TC and labeled as TGC (6 h UV light irradiation).

Other samples with irradiation of 2, 4, 12 h (donated as TGC-2, TGC-4, TGC-12 h) are also prepared.

Several other cupric salts [such as CuCl_2_, Cu(CH_3_COO)_2_, Cu(NO_3_)_2_] were employed to substitute CuSO_4_ to obtain final products labeled as TGC-Cl2, TGC-A, TGC-N (6 h UV light irradiation).

### Photoelectrochemical performance

The photoelectrochemical measurement was performed by a CHI 760E electrochemical workstation (Shanghai CH instrument Co., Ltd, China), with Pt plate as counter electrode, Ag/AgCl (filled with 3.5 M KCl aqueous solution) as reference electrode and 0.2 M Na_2_SO_4_ aqueous solution as electrolyte. The working electrode was prepared as follows: 10 mg product powder was mixed with 22 μL PVDF poly(vinylidene fluoride) solution, and that PVDF was dissolved in *N*-methyl-2-pyrrolidone (wt% 5%) with weight ratio of 90:10 to make slurry. The film was made by doctor blade method on FTO for area of 1×1 cm^2^, then vacuum-dried at 100 °C for 12 h. The uncovered area of FTO which would be immersed in electrolyte was protected by insulting glue.

### Photocatalytic performance

The photocatalytic performance was measured by photodegradation of MO, RhB and SDBS. In a typical process, 20 mg of photocatalysts and 100 mL MO/SDBS/RhB solution (20 mg L^−1^) were sonicated for 10 min to obtain homogeneous suspension. Before light irradiation, the suspension was stirred for 0.5 h in dark to achieve adsorption and desorption equilibrium. Then, 5 mL of the solution was extracted every 0.5 h for UV–Vis absorption measurement. The photoreaction was carried out in the protection of cycling cool water. The light source is 350 W Xenon lamp to simulate solar light (range of spectrum is from 200 to 2500 nm).

### Characterization

Powder X-ray diffraction (XRD) was performed on DX-2700 X-ray diffractometer (Dandong Fangyuan, China) with monochromatized Cu-K*α* radiation (*λ* = 1.5418 Å) at 40 kV and 30 mA. Transmission electron microscopy (TEM) images were taken with JEOL JEM-2100 transmission electron microscope at 200 kV. The concentration of MO was analyzed by measuring the light absorption at 484 nm UV–Vis 756PC Spectrophotometer (Shanghai Spectrum Instruments Co., Ltd. China). Fourier transform infrared (FTIR) spectra were obtained using BRUKER Tensor II spectrometer in the frequency range of 4000–400 cm^−1^ with a resolution of 4 cm^−1^. Measurement of Raman spectra was performed on a Raman DXR Microscope (Thermo Fisher, USA) with excitation laser beam wavelength of 532 nm. PL spectrum was measured at room temperature on a 7-PLSpec fluorescence spectrophotometer (Saifan, China). The wavelength of the excitation light is 325 nm. Optical absorption spectra were recorded on a UV–Vis spectrometer (UV-2600, Shimadzu, Japan) over a spectral range of 200–1400 nm. X-ray photoelectron spectroscopy (XPS, Thermo ESCALAB 250XI) with Al K*α* (*hv* = 1486.6 eV) radiation and beam spot of 500 μm was operated at 150 W. The Brunauer–Emmett–Teller (BET) surface areas were characterized by a surface area analyzer (Micromeritics, ASAP2020 M, USA) with nitrogen adsorption at 77 K.

## Results and discussion

### Characterization of phase and morphology

The XRD peaks of crystalline Cu_2_O were observed in Fig. S2a and 1 for TC and TGC, which indicated Cu_2_O (PDF#05-0667) could be synthesized under UV radiation directly without assistance of any chemical reagent at room temperature. Peaks of crystallized anatase TiO_2_ (PDF#21-1272) were observed in the TC and TGC samples. Graphene oxide (GO) fabricated by Hummers’ method in aqueous solution was reduced to rGO under the UV irradiation [31, 33, 34], which was also demonstrated by IR spectrum shown in Fig. 3a. However, no GO or rGO peak was found since ordered stacking of rGO sheets had been disrupted by loading TiO_2_ nanosheets and Cu_2_O [33].

**Figure 1:**
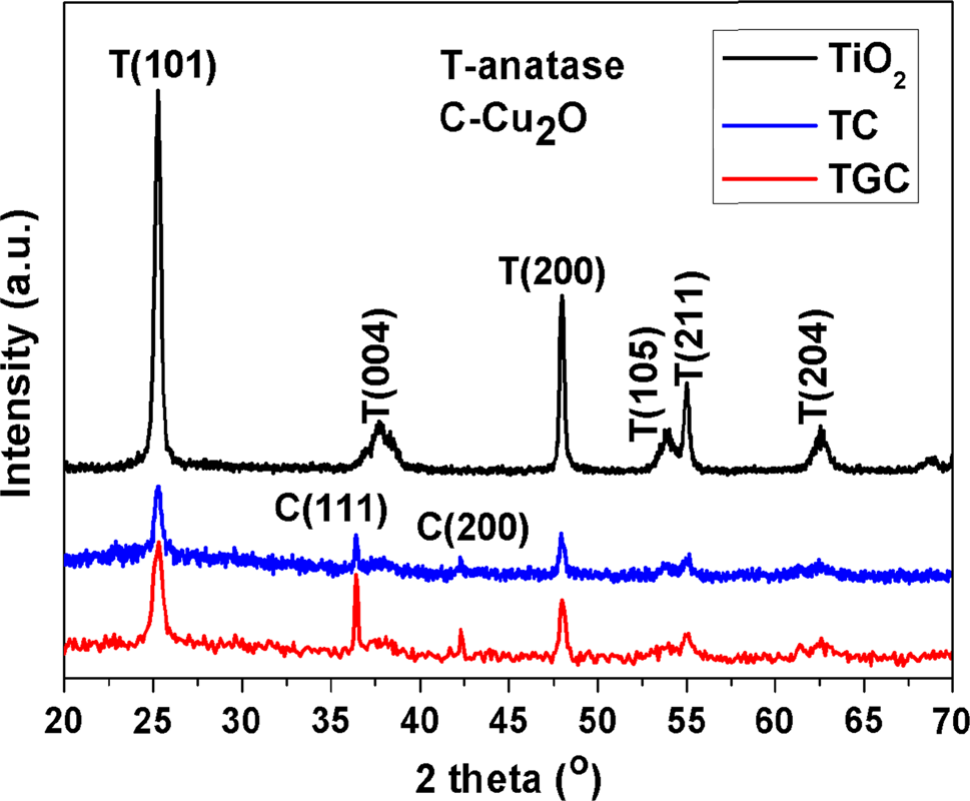
XRD patterns of TiO_2_, TC, TGC samples

**Figure 2:**
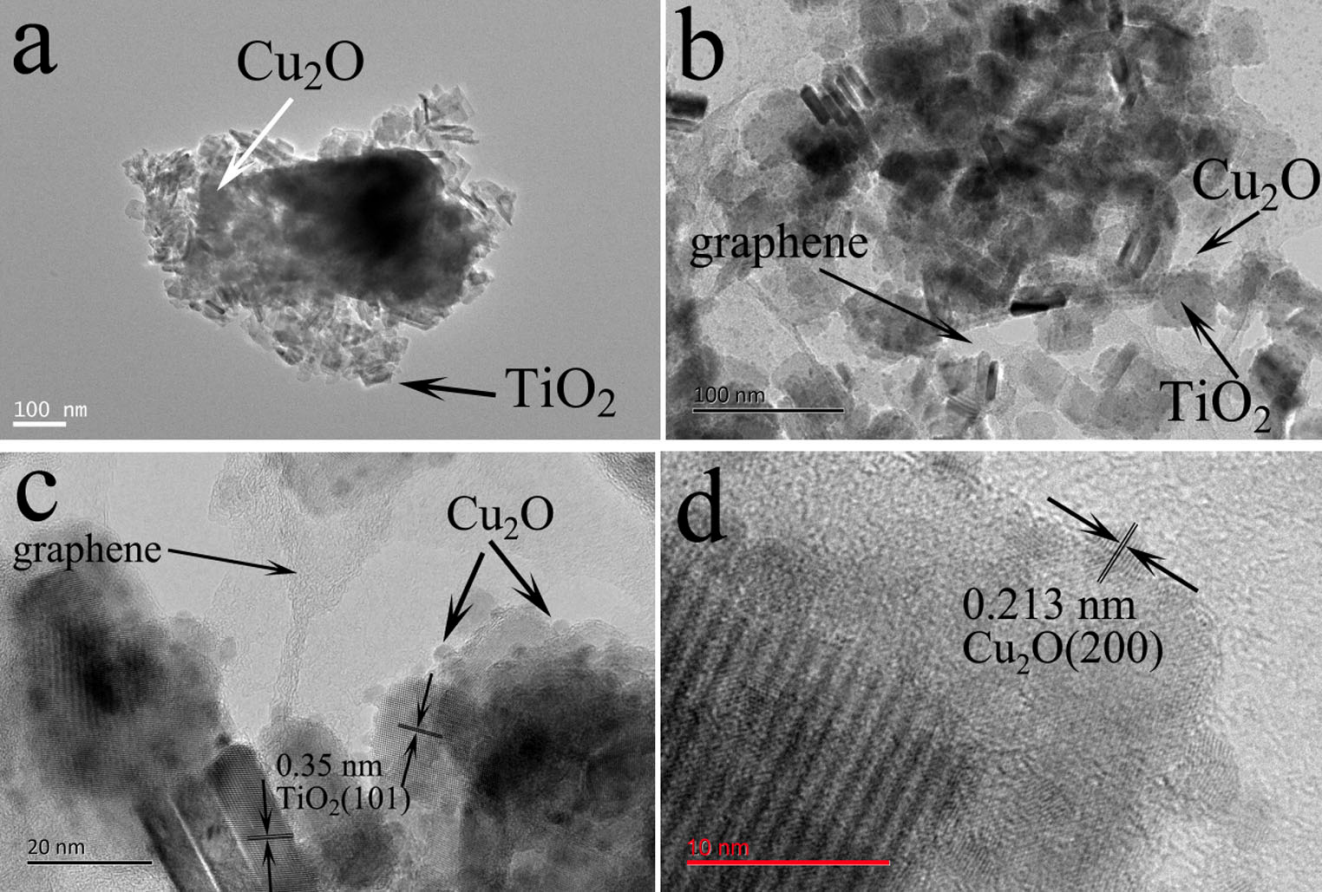
TEM of sample TC (**a**), TGC (**b**, **c**); HRTEM of Cu_2_O nanocrystals (**d**), displaying the (200) exposed facet, with lattice space of 0.213 nm

TiO_2_ nanosheets were prepared by the classical hydrothermal method [32], which had rectangular shape with the length of ca. 50 nm and thickness of 5 nm, as shown in Fig. S1. As Cu_2_O composited with TiO_2_ forming sample TC, large amounts of Cu_2_O nanocrystals were deposited on TiO_2_ nanosheets and even self-aggregated because of large quantities, as shown in Fig. 2a. After further compositing with rGO, it is observed that large amounts of Cu_2_O nanocrystal with size of ca. 5 nm adhered on TiO_2_ nanosheets and rGO sheet in Fig. 2b, c, which was ascribed to residual oxygen-containing groups of rGO facilitating dispersion of Cu^+^. This new morphology was achieved only via UV irradiation without addition of any chemical reducing reagent, so this work provides a novel way for synthesizing nano-Cu_2_O and its composites.

### Characterization of IR and Raman spectrum

**Figure 3:**
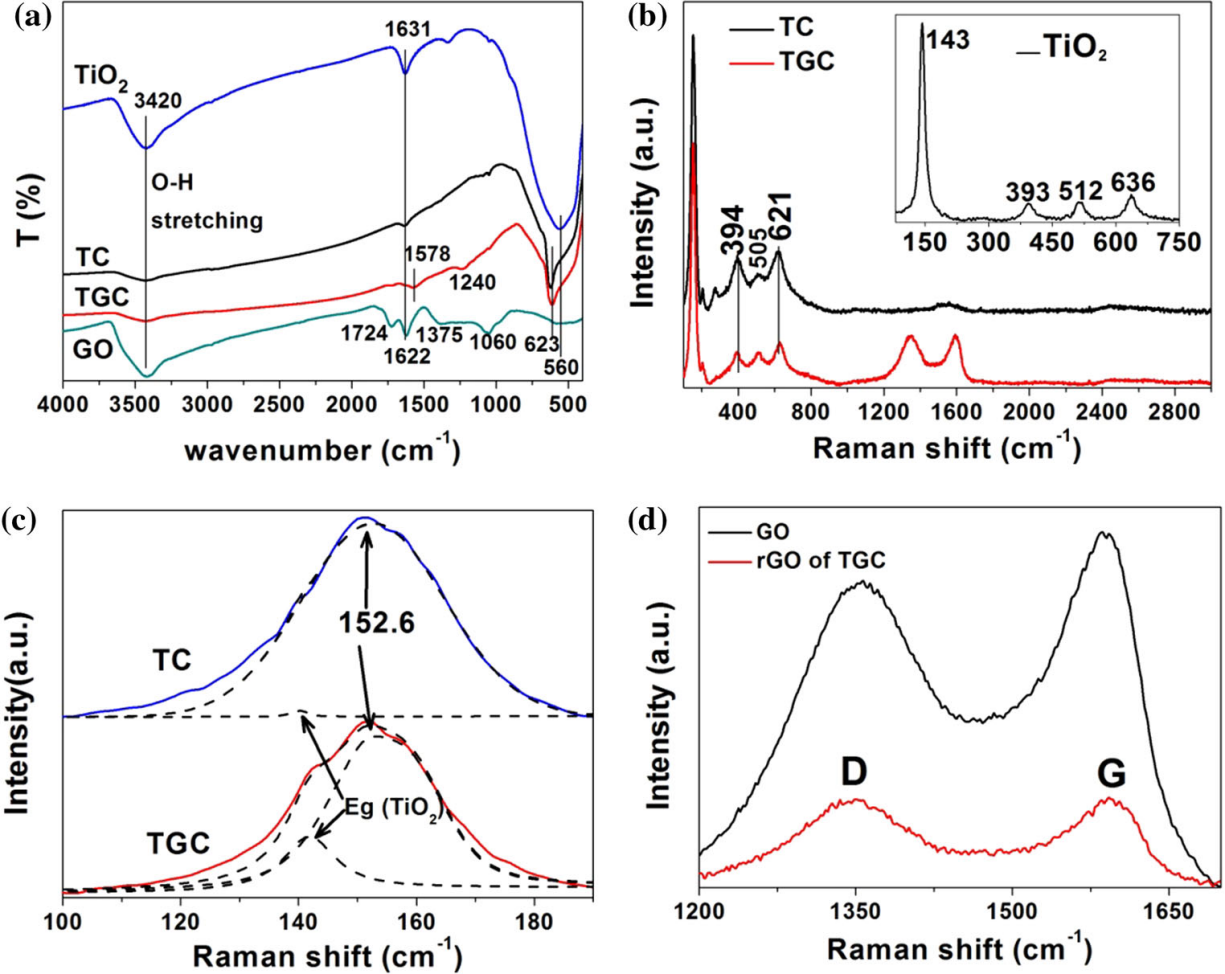
**a** FTIR spectrum of TiO_2_, TC, TGC, GO samples; **b** Raman spectrum of TC and TGC samples, the *inset* is anatase TiO_2_; **c** Fitting of Raman bands of TC and TGC by Lorentzian–Gaussian functions; **d** Raman spectra of rGO in TGC and GO

Chemical bond and phases of composites were characterized by FRIT and Raman spectrum (Fig. 3). In Fig. 3a, around 3420 cm^−1^ corresponded to the O–H stretching vibration of alcoholic or phenolic groups as well as intercalated or adsorbed water molecular for all samples [35–38]. The peak of 1631 cm^−1^ was attributed to bending mode of surface –OH or water for TiO_2_, TC [39]. The broadband around 560 cm^−1^ between 880 and 400 cm^−1^ showed the vibration of Ti–O–Ti bonds of sample TiO_2_, TC, TGC [40]. The sharp peak of 623 cm^−1^ was attributed to the stretching of copper(I)–O bond in TC and TGC, which indicated the formation of Cu_2_O [41]. In comparison with stretching modes of carbonyl (C=O) bond (1724 cm^−1^), conjugation absorption for bending mode of water and C=C *sp* ^2^ hybrid(1622 cm^−1^), tertiary alcohol (C–OH) bending (1375) and alkoxy (C–O) vibrations (1060) in IR spectrum of GO, most oxygen-containing groups of rGO in TGC were removed by the UV reduction [42, 43], its absorption peak of C=C bond was shifted to 1578 cm^−1^. The 1240 cm^−1^ peak should be attributed to stretching modes of the epoxy (C–O–C) group that can hardly removed by UV irradiation [44].

In Fig. 3b, four strong vibration peaks at 143 cm^−1^ (E_g_), 393 cm^−1^ (B_1g_), 512 cm^−1^ (A_1g_) and 615 cm^−1^ (E_1g_) were ascribed to the five Raman active modes (A_1g_ + B_1g_ + 3E_g_) of anatase [45]. The peaks around 120–180 cm^−1^ for TC and TGC were decomposed to the sharp peak at 152.6 cm^−1^, which should be mainly attributed to the Γ_15_^(1)^ (LO) infrared (ir)-allowed mode in perfect Cu_2_O crystals and a small peak for E_g_ mode of anatase as shown in Fig. 3c [46]. The peak at 505 and 621 cm^−1^ should be assigned to the overlapping of Raman vibration mode of crystalline Cu_2_O and TiO_2_ [45, 47]. The peak at 394 cm^−1^ was the B_1g_ mode of anatase TiO_2_ for TC and TGC. Phase determination of Raman spectra agreed with the results of XRD (Fig. 1). D band provided information about defect of graphitic structure and the presence of *sp* ^3^-hybridized domain [17]. G band was a prominent feature of the pristine graphite, corresponding to the first-order scattering of the E_2g_ mode [48]. In Fig. 3d, the position of D and G band of GO was about 1357 and 1586 cm^−1^, respectively. After reduction, the D and G band for rGO of TGC shifted to 1350, 1592 cm^−1^, respectively, and *I*_D_/*I*_G_ increased from 0.91 to 1.13 after UV reduction of GO, which indicated a decrease in average size and increase in numbers of the *sp* ^2^-hybridized domains of rGO in TGC, comparing with that of GO [48, 49].

### XPS analysis

**Figure 4:**
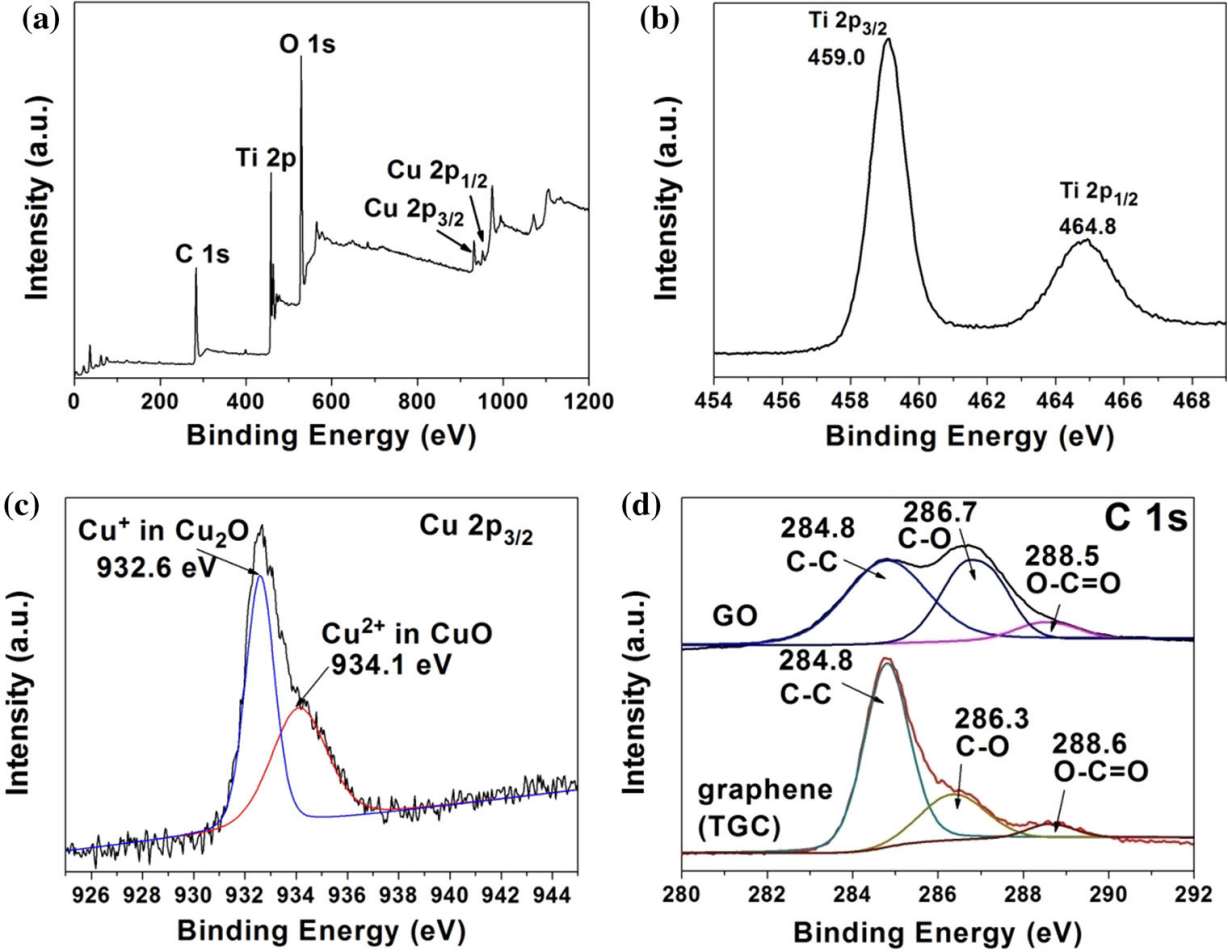
XPS spectra of TGC sample calibrated at C 1 s 284.8 eV, **a** full spectrum, **b** Ti 2p spectrum, **c** Cu 2p_3/2_ spectrum and **d** C 1 s for TGC and GO spectra

Figure 4a demonstrates the full spectrum of sample TGC, while Fig. 4b–d focuses on the specific binding energy of element Ti, Cu and C, respectively. The Ti 2*p* peaks located at the binding energies of 459.0 and 464.8 eV were attributed to Ti 2*p*_3/2_ and Ti 2*p*_1/2_, which corresponded to Ti^4+^ [50]. In Fig. 4c, the peak for Cu 2*p*_3/2_ was decomposed into two peaks, the main peak of which at 932.6 eV was the characteristic of Cu^+^ in Cu_2_O [28], and the peak at 934.1 eV indicated the existence of Cu^2+^ in CuO [51]. XPS could only detect the shallow surface elements composition, so the observation of Cu^2+^ indicated the oxidation of a small portion of Cu_2_O during sample drying and handing under normal ambient condition. This phenomenon had been reported by many researchers on the synthesis of Cu_2_O nanoparticles. From C 1*s* XPS spectrum of GO (Fig. 4d), the peaks at 282.8, 286.7, 288.5 eV were assigned to the *sp* ^2^-hybrid bond (C–C, C=C, C–H), C–O and O–C=O bond, respectively [52]. After UV reduction, a large amount of C–O and O–C=C bonds were removed as indicated by the decrease in intensities of these two peaks, which was consistent with results of IR spectra.

### Selective adsorption and photocatalytic performance

**Figure 5:**
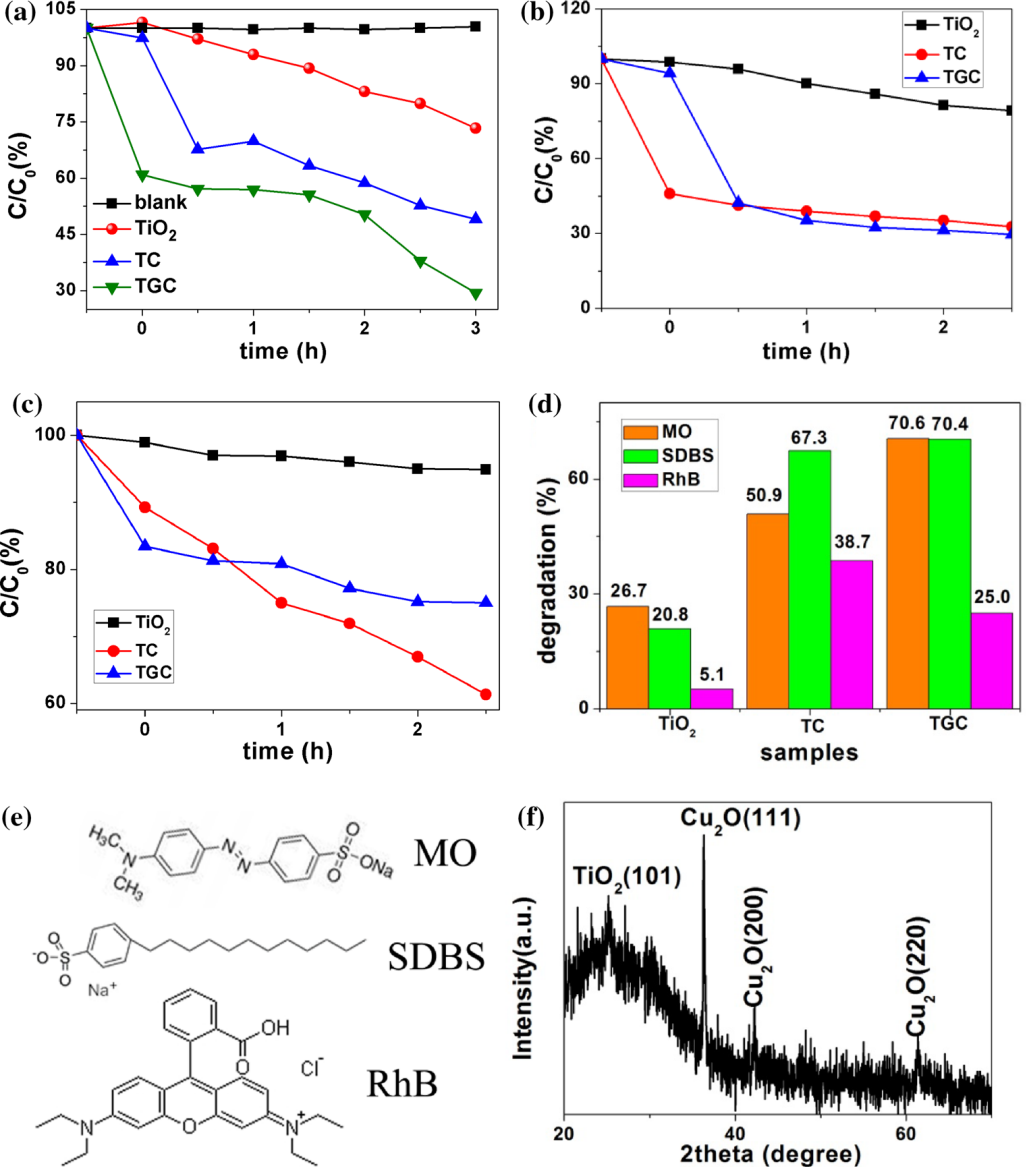
Photocatalytic degradation performance of **a** MO, **b** SDBS and **c** RhB, under irradiation of 350 W Xenon lamp simulating solar light AM1.5; **d** comparison of photodegradation for MO, SDBS and RhB; **e** molecule structure of MO, SDBS and RhB; **f** XRD pattern of TGC after 12 h under simulated solar light

Photocatalytic performances of samples were characterized by degradation of MO, SDBS and RhB (Fig. 5a–c). All experiments were carried out in nearly neutral solution, shown in Table S1. The MO is no self-degradation. Pure TiO_2_ nanosheets had the lowest degradation ability for 26.7%, while sample TC had stronger degradation ability (50.9%) after compositing with Cu_2_O. Further incorporation of rGO can facilitate MO adsorption, and thus, the TGC had highest photocatalytic activity for MO degradation (70.6%). For SDBS degradation, TiO_2_ had lowest photocatalytic activity (20.8%). Sample TGC had slightly better activity than TC (ca. 70%). For RhB degradation, TiO_2_ still had poorest activity than others because of its absorbance limitation of solar light. TC had better photocatalytic performance than TGC. As shown in Fig. 5d, within 2.5 h, MO and SDBS could be decomposed dramatically by TC and TGC under solar light; however, the degradation efficiency for RhB was much lower than that for other contaminations, which indicated that only some special contaminations could be decomposed by nanoheterojunction effectively.

The adsorption of photocatalysts for contaminations played an important role in the process of photocatalytic reactions. Contaminants owning opposite charge with the photocatalysts could easily adsorb on the surface of photocatalysts preferentially. However, given that the charge of MO/SDBS/RhB (molecule structures are shown in Fig. 5e) in aqueous solution and adsorption abilities of the synthesized photocatalysts, the reverse charge principle was not perfect for explaining adsorption difference. Taken some special groups of contaminants into consideration, nitrogen-containing groups may also affect the eventual adsorption outcome.

Chemical stability of Cu_2_O particles is one of predominant factors for photochemical applications. Exposed to UV light (irradiated by varied lamp), Cu_2_O can be reduced to metal Cu [53, 54]. However, in this work under standard simulated solar light, Cu_2_O was very stable and no metal copper was found even after 12 h with the characterization of X-ray diffraction (Fig. 5f).

### Photocatalytic mechanism

**Figure 6:**
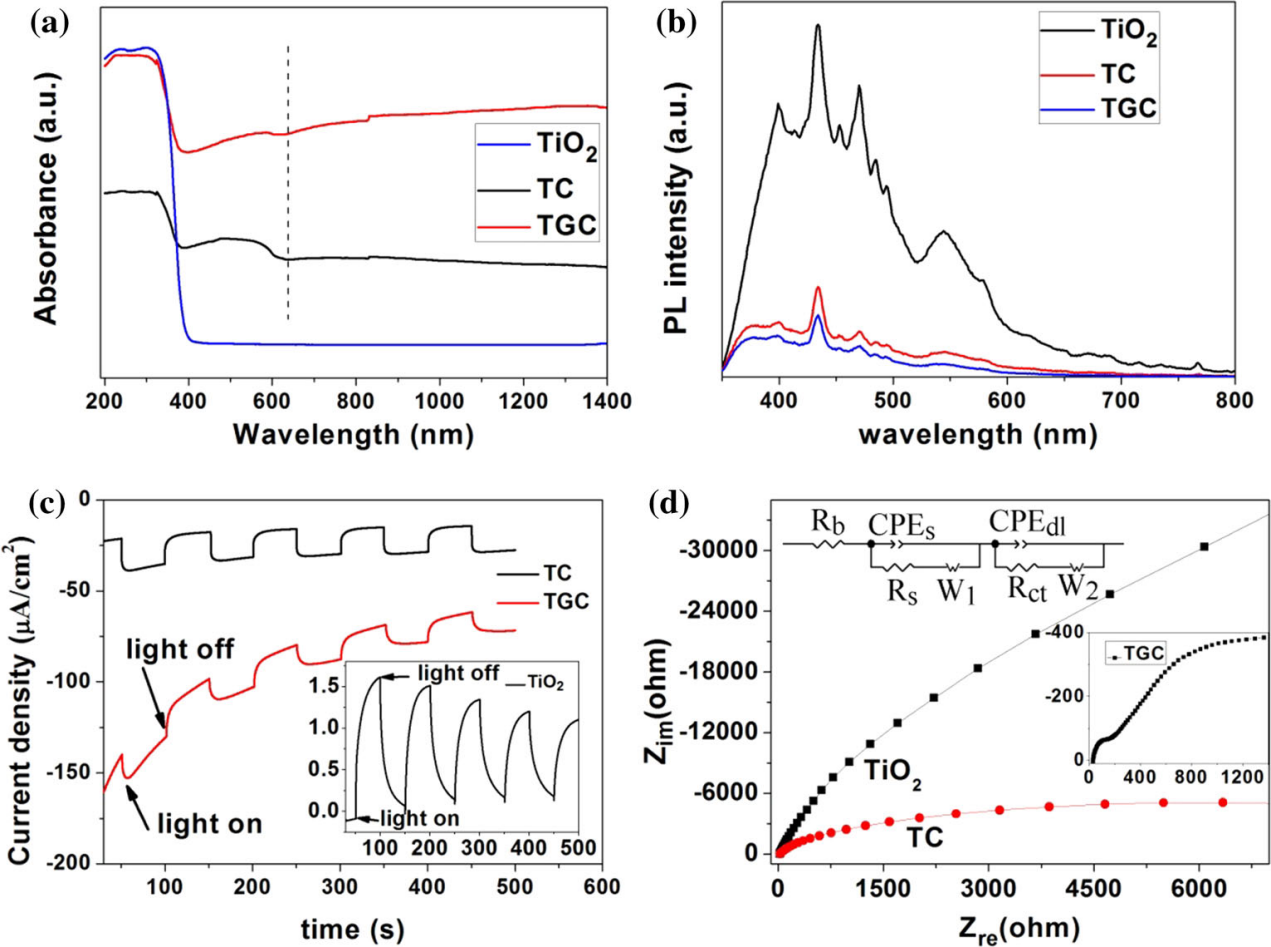
**a** UV–Vis diffuse reflectance spectra of TiO_2_, TC, TGC; **b** PL spectrum of TiO_2_, TC, TGC; **c** photocurrent curve of TiO_2_ (*inset*), TC and TGC under simulated solar light; **d** electrochemical impedance spectrum (EIS) of TiO_2_, TC and TGC (*inset*)

As shown in Fig. 6a, TiO_2_ could only absorb UV light below 387 nm. Cu_2_O enhanced absorption of visible light below 640 nm in TC. Moreover, with incorporation of rGO, TGC had highest light absorbance, so it induced more photo-induced charge carriers and higher degradation rate. Optical band gap of composite was calculated using Tauc plot shown in Fig. S3d [55]. Comparing with pure TiO_2_ (3.2 eV), optical band gap of TC and TGC decreased to 2.72 and 2.64 eV, respectively.

PL spectrum was employed to characterize separation efficiency of photo-generated electron–hole pairs. As shown in Fig. 6b, several peaks such as 400, 434, 470, 544 nm were observed in PL spectrum of TiO_2_, which attributed to electron transition from the conduction band to valence band, band-edge free excitons, oxygen vacancies or surface defect [56, 57]. TC had lower intensity of PL than pure TiO_2_ that inferred Cu_2_O could accept the photocharge from TiO_2_. Furthermore, lowest intensity of TGC demonstrated that rGO could further accept the photo-induced electron and enhance separation efficiency of electron–hole pairs.

TiO_2_ electrode in Na_2_SO_4_ aqueous solution had a positive photocurrent, indicating its n-type semiconductor nature, shown in inset of Fig. 6c. Oppositely, sample TC and TGC exhibited a negative current response and larger photocurrent, which was a sign of p-type semiconductor of Cu_2_O (also demonstrated in Fig. S2b). In addition, Cu_2_O could also enhance the charge transportation in TC, compared to the current baseline of TiO_2_. The rGO further enhanced the conductivity of TGC. As shown in Fig. 6d, the typical electrochemical impedance spectra were presented as Nyquist plots. For fitting the EIS, equivalent circuit [model: R(Q(RW))(Q(RW))] was demonstrated that the simulating results fitted the experimental very well. Q is constant phase element (CPE). *R*_b_ represented the bulk resistance, CPEs should be considered in the nonhomogeneous condition of the composites, associating with the capacitor, and *R*_s_ are the resistance of the solid-state interface layer which is formed at the highly charged state due to the passivation reaction between the electrolyte and the surface of the electrode, corresponding to the first semicircle at high frequency [58]. CPE_dl_ and *R*_ct_ are the double-layer capacitance and the charge-transfer resistance, corresponding to the second semicircle at medium frequency. Nyquist plots of EIS showed that nanocrystalline Cu_2_O could decrease the *R*_ct_ from 9.4×10^5^ to 1.2×10^4^ ohm cm^2^ for TC because of the smaller semicircle at the medium frequency, in comparison with TiO_2_ (Fig. 6d) [40]. The resistance of TGC dramatically decreased to 4.4 ohm cm^2^ because of high conductivity of rGO as shown in inset of Fig. 6d. Its high charge shuttle and transfer enhanced the degradation ability.

**Figure 7:**
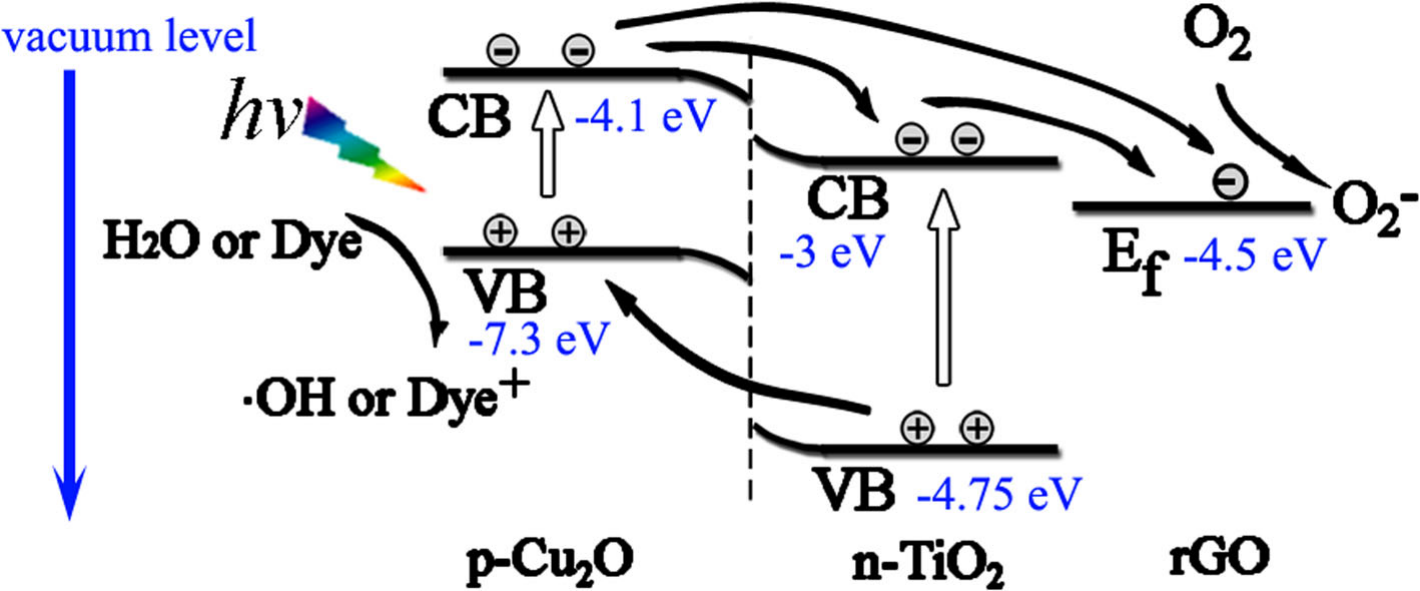
Illustration of transfer of photo-generated electron–hole pairs excited by solar light. The dissolved O_2_ scavenge the photo-generated electron transferred from Cu_2_O or TiO_2_ to form O_2_^−^, the photohole oxidized H_2_O to OH or decompose dye directly. Band gaps of TiO_2_ and Cu_2_O were determined by UV–Vis diffuse reflection spectroscopy. Energy levels referred to Refs. 22, 59–61

Photocatalytic performance of TGC was the best in the photocatalysts for MO and SDBS degradation. The schematic illustration is shown in Fig. 7: (1) The band gap of Cu_2_O determined by UV–Vis diffuse reflection spectroscopy (Fig. S3b) is 1.75 eV, which could enhance visible light absorption below 708 nm, generating more electron–hole pairs; (2) the matching energy band structure facilitated the separation of electron–hole pairs of heterojunction [15, 29, 62]; (3) rGO facilitated the dispersion of nanocrystals, transfer and shuttle of photo-generated electron in metal oxide particles [63].

### Reduction mechanism of TGC via UV

**Figure 8:**
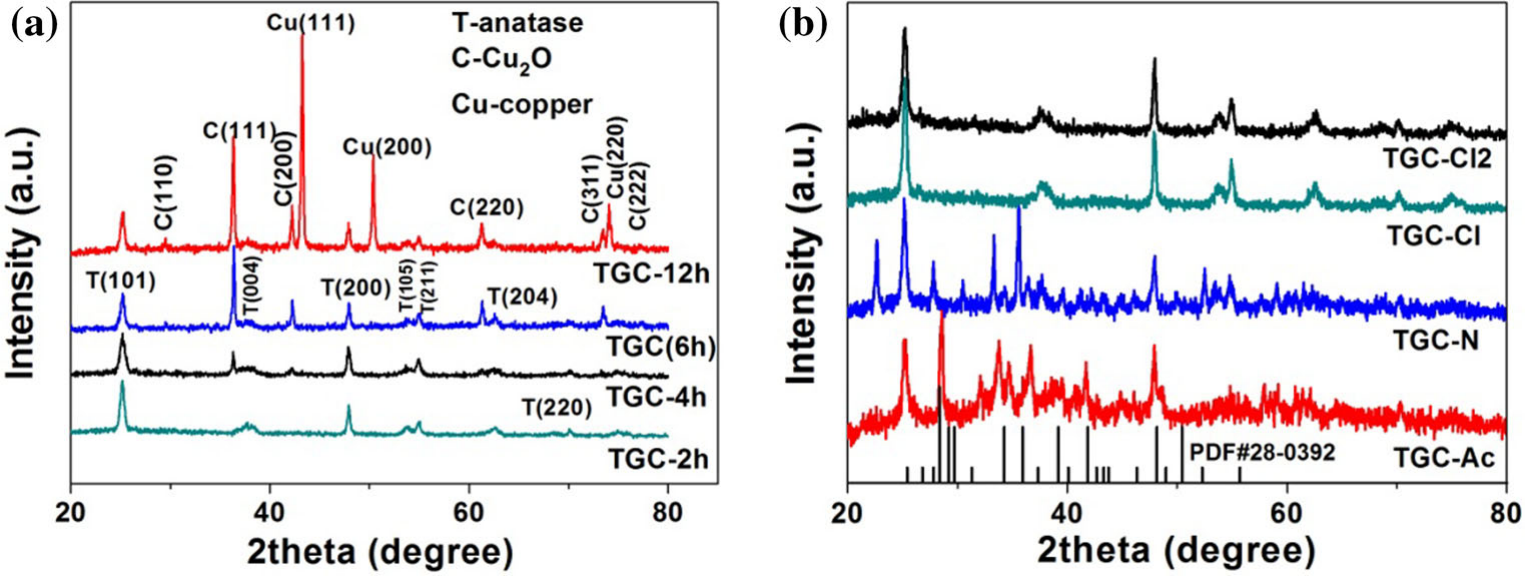
XRD patterns of **a** TGC-2 h, TGC-4 h, TGC (6 h), TGC-12 h and **b** TGC-A, TGC-N, TGC-Cl2, TGC-Cl

Figure 8a shows the effect of irradiation time on phase of products. No Cu_2_O could be detected by the XRD after 2-h reaction. If the reaction prolonged for 4 h, a weak peak (111) of Cu_2_O could be observed, then more Cu_2_O were synthesized after 6 h. However, when the UV reduction time extended to 12 h, part of Cu_2_O particle converted to metal Cu.

Several other cupric salts [such as CuCl_2_, Cu(CH_3_COO)_2_, Cu(NO_3_)_2_] were tried to synthesize Cu_2_O products, labeled as TGC-Cl2, TGC-A, TGC-N (other synthesizing conditions were the same as that of sample TGC). There was CH_3_COOCu (PDF#28-0392) formed under UV reduction with (CH_3_COO)^−^ involvement, shown in Fig. 8b. As NO_3_^−^ used, the final phases of TGC-N could not be identified at present. As Cl^−^ used, all XRD peaks for TGC-Cl2 were assigned to anatase and no copper-containing phase was detected. Furthermore, if NaCl was added to the TGC synthesis process, no diffraction peaks of copper-containing phase were found either (shown in Fig. 8b donated as TGC-Cl). It demonstrated that Cl^−^ could chelate Cu^+^ preferentially instead of OH^−^.

Above all, the mechanism of synthesizing Cu_2_O could be proposed as follows:

$$ \begin{array}{*{20}l} {{\text{H}}_{2} {\text{O}}\mathop{\longrightarrow}\limits^{hv}{\text{H}}^{ + } + {\text{H}} {\cdot} + {\cdot} {\text{OH}} + {\text{e}}_{\text{aq}}^{ - } \ldots {\text{etc}}} \hfill \\ { {\cdot} {\text{OH}} + {\text{CH}}_{3} {\text{CH}}_{2} {\text{OH}} \to {\text{CH}}_{3} \dot{\text{C}} {\text{OH}} + {\text{H}}_{2} {\text{O}}} \hfill \\ {{\text{Cu}}^{2 + } + {\text{e}}_{\text{aq}}^{ - } \to {\text{Cu}}^{ + } } \hfill \\ {{\text{Cu}}^{ + } + {\text{OH}}^{ - } \to {\text{CuOH}}} \hfill \\ {2{\text{CuOH}} \to {\text{Cu}}_{2} {\text{O}} + {\text{H}}_{2} {\text{O}}} \hfill \\ \end{array} $$

If extending irradiation time, there would be:

$$ {\text{Cu}}_{2} {\text{O}} + 2{\text{e}}^{ - } + {\text{H}}_{2} {\text{O}} \to 2{\text{Cu}} + 2{\text{OH}}^{ - } $$

If CH3COO^−^ was employed as the precursor:

$$ \begin{array}{*{20}l} {{\text{Cu}}^{2 + } + {\text{e}}_{\text{aq}}^{ - } \to {\text{Cu}}^{ + } } \hfill \\ {{\text{Cu}}^{ + } + {\text{OH}}^{ - } + {\text{CH}}_{3} {\text{COO}}^{ - } \to {\text{CH}}_{3} {\text{COOCu}} + {\text{OH}}^{ - } } \hfill \\ \end{array} $$

If Cl^−^ was added into the reaction system of TGC, the reaction was changed to:

$$ \begin{array}{*{20}l} {{\text{Cu}}^{2 + } + {\text{e}}_{\text{aq}}^{ - } \to {\text{Cu}}^{ + } } \hfill \\ {{\text{Cu}}^{ + } + {\text{OH}}^{ - } + {\text{Cl}}^{ - } \to {\text{CuCl}}_{\text{aq}} + {\text{OH}}^{ - } } \hfill \\ \end{array} $$

So compared to CH_3_COO^−^/NO_3_^−^/Cl^−^, SO_4_^2−^ is the optimum anion for UV reduction synthesis of Cu_2_O.

## Conclusion

Pure Cu_2_O, TiO_2_/Cu_2_O, TiO_2_/rGO/Cu_2_O nanoheterojunctions were fabricated by novel UV reduction method, and large amounts of dot-like Cu_2_O nanocrystals with size of ca. 5 nm were formed on the rGO or TiO_2_ nanosheets. Sample TGC achieved the strongest absorption for solar light, highest separation efficiency of photo-induced electron–hole pairs. It had p-type photocurrent response under solar light and excellent photocatalytic performance. The adsorption abilities for catalysts varied with different dyes or surfactant, determined by nitrogen-containing groups and surface charge. Extending irradiation time could convert Cu_2_O to metal copper. In comparison with CH_3_COO^−^/NO_3_^−^/Cl^−^, SO_4_^2−^ is the optimum anion for synthesis of pure Cu_2_O phase under UV condition.

## Electronic supplementary material

Below is the link to the electronic supplementary material.

Supplementary material 1 (DOCX 16252 kb)
