# Supplementary material for: Photocatalytic performance of Cu2O-loaded TiO2/rGO nanoheterojunctions obtained by UV reduction
Source: J Mater Sci. 2017 Feb 21;52(11):6754–66. doi: 10.1007/s10853-017-0911-2 (PMC5348572; doi:10.1007/s10853-017-0911-2)
Supplement: Supplementary file 1 — Supplementary material 1 (DOCX 16252 kb) [file 10853_2017_911_MOESM1_ESM.docx]

Supporting information

Photocatalytic performance of Cu₂O loaded TiO₂/ rGO nano-heterojunctions obtained by UV-reduction

Kaituo Dong^a^*, Jiandong He^a^, Junxue Liu^b,c^, Fengting Li^a^, Lianqing Yu^a^*, Yaping Zhang^a^, Xiaoyan Zhou^a^, Hongzhang Ma^a^

a College of Science and Key Laboratory of New Energy Physics and Materials Science, China University of Petroleum, QingDao 266580, China

b College of Chemical Engineering, China University of Petroleum, QingDao 266580, China

c State Key Laboratory of Molecular Reaction Dynamics, Dalian Institute of Chemical Physics, Chinese Academy of Sciences, 457 Zhong Shan Rd., Dalian, China, 116023

Synthesis of pure Cu_2_O

3 mmol CuSO_4_·5H_2_O was dissolved in 100 mL deionized water and 100 mL ethanol was added with fiercely stirring in the quartz concial flask. After vacuum pumping, the solution was exposed under UV light (the main peak at 254 nm, 25W) for 6 h with the protection of Ar. The final product was centrifuged and washed by water and ethanol several times, before vacuum-dried at 60 ℃ for 12 h.


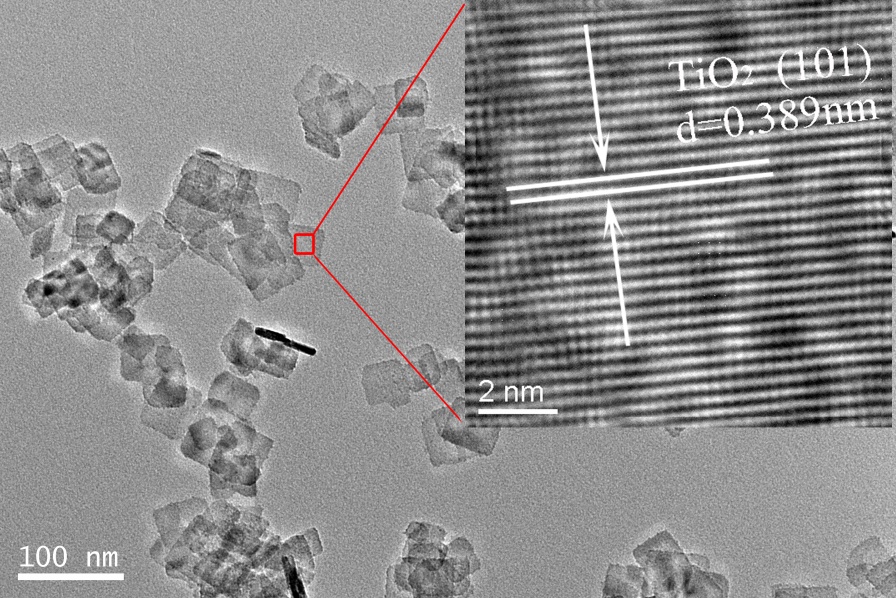


Fig. S1 TEM image of TiO_2_ nanosheets, the insert was the fourier transform of HRTEM of the red rectangle area and the space of crystal plane (101) was 0.389 nm;







Fig. S2 (a) XRD pattern of pure Cu_2_O; (b) current response of Cu_2_O film under solar light radiation in three electrodes (the Cu_2_O powder coated on the FTO as the working electrode, Pt plate as the counter electrode, Ag/AgCl as the reference electrode). The cathodic photocurrent demonstrates Cu_2_O synthesized by the UV light is p-type semiconductor.











Fig. S3 (a) UV-vis diffuse reflectance spectra of Cu_2_O; (b) plots of (A*hv*)^2^ versus *hv* for Cu_2_O; (c) plots of (A*hv*)^1/2^ versus *hv* for Anatase TiO_2_. "A" in the Y axis represents measured absorbance, which is proportional to the absorption coefficient (ɑ); (d) Tauc plot for band gap calculation of TiO_2_, TC, TGC

UV-visible diffuse reflection spectroscopy could characterize absorption of photocatalyst for solar light and the optical band gap of semiconductors. The optical band gap can be determined by the equation (1) [1,2]:

(ɑ*hv*)^n^=k(*hv*-E_g_) (1)

Which ɑ is the absorption coefficient, n is 2 for direct transition (for p-type Cu_2_O) [3], and 1/2 for indirect transition (for anatase) [4], k is a constant. *hv* is the photon's energy, Eg is the optical band gap. According to equation (1) the band gap of TiO_2_ was 3.2 eV, band gap of Cu_2_O decreased to 1.75 eV, which was different from the normal value (1.9~2.0 eV) as Fig. S3 (b)(c) showed [5]. The decreased band gap of Cu_2_O was resulted from increase of absorption from near infrared light of 1300 nm to visible light of 640 nm (shown in Fig. S3(a)), which might be ascribe to the defect, surface states, particle size distribution, indirect transition, existence of large amounts of amorphous Cu_2_O or tail of metal copper in the prepared Cu_2_O powders [6].

Table S1 pH of contamination solution added by photocatalysts

|  | RhB | SDBS | MO |
| --- | --- | --- | --- |
| none | 6.0 | 6.5 | 5.8 |
| TiO_2_ | 6.5 | 6.5 | 5.7 |
| TC | 6.5 | 6.5 | 5.7 |
| TGC | 6 | 6.3 | 5.8 |

BET analysis

Table S2 the BET surface area, pore volume and average pore size of TiO_2_, TC, TGC

|  | Specific surface area (m^2^/g) | Pore volume (cm^3^/g) | Average pore size (nm) |
| --- | --- | --- | --- |
| TiO_2_ | 94.6 | 0.37 | 15.5 |
| TC | 60.6 | 0.23 | 5.4 |
| TGC | 85.5 | 0.28 | 13.1 |







Fig. S4 N_2_ absorption–desorption isotherms of pure TiO_2_, TC, TGC; (b) Pore size distributions of pure TiO_2_ nanosheets, TC, TGC. Inset indicated the pore size, range of 2~6 nm

Fig. S4(a) showed all products had isotherms of type IV (IUPAC classification) with a broad hysteresis loop at relative pressure (P/P_0_) between 0.5 and 1.0, indicating the presence of mesopores and macropores [7]. The corresponding hysteresis loops are type H3 at a high relative pressure, range of 0.5 to 1.0, suggesting the presence of slit-like pores, which were generated by the aggregation of TiO_2_ nanosheets [8]. The hysteresis loop of TGC had the extended range from 0.5 to 1.0, comparing with those of two others from 0.8 to 1.0. The extended range generally demonstrated increase of mesopores adsorption, which agreed with their pore properties (see Table S2). Fig. S4(b) showed the pore distribution, derived from adsorption branch. All products showed a wide pore distribution from 2 nm to 50 nm. As for TiO_2_ nanosheets, pore diameter mainly distributes to 2.5 nm and 22 nm; as for TC and TGC, pore diameter distributes very dispersively, especially for TGC.

Table S2 showed specific surface area, pore volume of samples. After depositing Cu_2_O nanocrystals, surface area and pore volume of TC decreased to 60.6 m^2^/g and 0.23 cm^3^/g, comparing to that of TiO_2_ nanosheets (94.6 m^2^/g, 0.37 cm^3^/g), which ascribed to the fill of Cu_2_O nanocrystals in the pore of TiO_2_ nanosheets. Incorporating reduced graphene into TiO_2_/Cu_2_O system increased surface area to 85.5 m^2^/g and pore volume to 0.28 cm^3^/g.

reference

1. Butler MA (1977) Photoelectrolysis and physical properties of the semiconducting electrode WO_2_  Journal of Applied Physics 48 (5):1914-1920. doi:doi:http://dx.doi.org/10.1063/1.323948

2. Cheng H, Huang B, Dai Y, Qin X, Zhang X (2010) One-Step Synthesis of the Nanostructured AgI/BiOI Composites with Highly Enhanced Visible-Light Photocatalytic Performances. Langmuir 26 (9):6618-6624. doi:10.1021/la903943s

3. Tan Y, Xue X, Peng Q, Zhao H, Wang T, Li Y (2007) Controllable Fabrication and Electrical Performance of Single Crystalline Cu_2_O Nanowires with High Aspect Ratios. Nano Letters 7 (12):3723-3728. doi:10.1021/nl0721259

4. Chen X, Mao SS (2007) Titanium Dioxide Nanomaterials:  Synthesis, Properties, Modifications, and Applications. Chemical Reviews 107 (7):2891-2959. doi:10.1021/cr0500535

5. McShane CM, Siripala WP, Choi K-S (2010) Effect of Junction Morphology on the Performance of Polycrystalline Cu_2_O Homojunction Solar Cells. The Journal of Physical Chemistry Letters 1 (18):2666-2670. doi:10.1021/jz100991e

6. Zhao XK, Fendler JH (1991) Size quantization in semiconductor particulate films. The Journal of Physical Chemistry 95 (9):3716-3723. doi:10.1021/j100162a051

7. S.J. Gregg KSWS (1982) Adsorption, Surface Area and Porosity. Academic Press, London

8. Sing KSW (1985) Reporting physisorption data for gas/solid systems with special reference to the determination of surface area and porosity (Recommendations 1984). vol 57. Pure and Applied Chemistry. doi:10.1351/pac198557040603
